# Dravet Variant *SCN1A*^*A*1783*V*^ Impairs Interneuron Firing Predominantly by Altered Channel Activation

**DOI:** 10.3389/fncel.2021.754530

**Published:** 2021-10-28

**Authors:** Nikolas Layer, Lukas Sonnenberg, Emilio Pardo González, Jan Benda, Ulrike B. S. Hedrich, Holger Lerche, Henner Koch, Thomas V. Wuttke

**Affiliations:** ^1^Department of Neurology and Epileptology, Hertie Institute for Clinical Brain Research, University of Tübingen, Tübingen, Germany; ^2^Institute for Neurobiology, Eberhard Karls University Tübingen, Tübingen, Germany; ^3^Bernstein Center for Computational Neuroscience, Eberhard Karls Universitat, Tübingen, Germany; ^4^Department of Epileptology, Neurology, Rheinisch-Westfälische Technische Hochschule Aachen, Aachen, Germany; ^5^Department of Neurosurgery, University of Tübingen, Tübingen, Germany

**Keywords:** Dravet syndrome (SMEI), developmental epileptic encephalopathy, SCN1A, Na_v_1.1, *in silico* modeling, electrophysiology

## Abstract

Dravet syndrome (DS) is a developmental epileptic encephalopathy mainly caused by functional Na_V_1.1 haploinsufficiency in inhibitory interneurons. Recently, a new conditional mouse model expressing the recurrent human p.(Ala1783Val) missense variant has become available. In this study, we provided an electrophysiological characterization of this variant in tsA201 cells, revealing both altered voltage-dependence of activation and slow inactivation without reduced sodium peak current density. Based on these data, simulated interneuron (IN) firing properties in a conductance-based single-compartment model suggested surprisingly similar firing deficits for Na_V_1.1^A1783V^ and full haploinsufficiency as caused by heterozygous truncation variants. Impaired Na_V_1.1^A1783V^ channel activation was predicted to have a significantly larger impact on channel function than altered slow inactivation and is therefore proposed as the main mechanism underlying IN dysfunction. The computational model was validated in cortical organotypic slice cultures derived from conditional *Scn1a*^*A*1783*V*^ mice. Pan-neuronal activation of the p.Ala1783V *in vitro* confirmed a predicted IN firing deficit and revealed an accompanying reduction of interneuronal input resistance while demonstrating normal excitability of pyramidal neurons. Altered input resistance was fed back into the model for further refinement. Taken together these data demonstrate that primary loss of function (LOF) gating properties accompanied by altered membrane characteristics may match effects of full haploinsufficiency on the neuronal level despite maintaining physiological peak current density, thereby causing DS.

## Introduction

Dravet syndrome (DS) is a developmental epileptic encephalopathy (DEE) with early childhood febrile seizures followed by a high-frequency seizure period with a large diversity of seizure types (Dravet, 1978). The epilepsy phenotype is accompanied by a multitude of comorbidities, including intellectual disability, sleep disorder, motor dysfunction, and an increased incidence of sudden unexpected death in epilepsy. More than 80% of diagnosed Dravet cases are caused by *de-novo* loss of function (LOF) variants causing functional haploinsufficiency of the *SCN1A* gene coding for the alpha-subunit of the voltage-gated sodium channel Na_V_1.1 (Marini et al., 2011). *SCN1A* variants are associated with multiple neurological diseases including generalized epilepsy with febrile seizures plus, sporadic/familial hemiplegic migraine, and DS resembling a clinical phenotype at the severe end of the spectrum (Gambardella and Marini, 2009). The high diversity of *SCN1A* associated disorders can be explained by a variety of gain and loss of function variants (Catterall et al., 2010), which impair the channel on different levels including transcriptional changes (Lange et al., 2019), reduced protein expression, altered membrane trafficking (Thompson et al., 2012), impaired ß-subunit interaction (Spampanato et al., 2004), and channel gating dysfunction (Kluckova et al., 2020).

In the past years, different cell cultures and animal models have been established based on patient variants to explore the different pathomechanisms of Na_V_1.1. Most frequently, heterozygous Na_V_1.1 knockout mice, mimicking protein-truncating non-sense variants found in patients with DS, have been used to investigate pathophysiological disease mechanisms (Yu et al., 2006; Ogiwara et al., 2007; Mistry et al., 2014; Favero et al., 2018). Such studies revealed a predominant role of LOF effects in certain inhibitory neuron classes including fast-spiking interneurons (FS-INs) (Bechi et al., 2012; Tai et al., 2014; Rubinstein et al., 2015; Favero et al., 2018; Tiraboschi et al., 2020; Tran et al., 2020). However, non-sense mutations only account for a subset of pathogenic variants leading to epileptic phenotypes. Especially milder epilepsy syndromes are often caused by missense variants within the protein-coding sequence and were studied in IPSC-derived neurons (Xie et al., 2020) and mouse models (Hedrich et al., 2014; Das et al., 2021). However, DS may also be caused by missense variants (Marini et al., 2011). Although protein truncation (non-sense variants) or diminished surface expression (certain missense variants) result in a pronounced reduction of sodium peak current, the mechanisms of remaining missense variants can be more complex, as physiological gating properties of channels may be affected on multiple levels. Although full haploinsufficiency is modeled well by heterozygous *Scn1a* knockout mice or knockin of a truncating variant (Yu et al., 2006; Ogiwara et al., 2007), they may not generally reflect the disease status of patients with DS for carriers of missense variants. To approach this uncertainty, conditional mice expressing the recurrent human Dravet missense variant p. Ala1783Val (A1783V) (Depienne et al., 2009; Klassen et al., 2014) have recently been developed by the Dravet Syndrome European Federation (Ricobaraza et al., 2019). A limited number of studies provide the first insight into the effects of mutant *Scn1a*^*A*1783*V*^ channels on neuron excitability after breeding conditional *Scn1a*^*A*1783*V*^ mice with Cre-driver lines. Depending on the exact genetic background of offspring animals, the subtype of studied neurons and the brain areas in which neurons were recorded, and the differential effects on neuron firing have been described (Kuo et al., 2019; Almog et al., 2021). Although LOF of Na_V_1.1^A1783V^ has been assumed, the biophysical effects of the variant on Na_V_1.1 channel function have not yet been delineated to date. In this study, we provided a detailed comparative characterization of variant p.(Ala1783Val) and wild type (WT) gating properties in tsA201 cells. Although full haploinsufficiency with reduced Na^+^ peak currents is a common feature in DS, our recordings revealed clear LOF mechanisms, albeit with preserved overall sodium peak current density. Building on these data, a single-compartment computational neuron model was developed to predict firing deficits in inhibitory and excitatory neurons for p.Ala1783Val in comparison to full haploinsufficiency by heterozygous knockout. The model was electrophysiologically validated in cortical organotypic brain slice cultures derived from *Scn1a*^*A*1783*V*^ mice. Interestingly, despite overall normal sodium peak current density, p.(Ala1783Val) was predicted to cause a fairly strong Interneuron (IN) firing deficit comparable to the heterozygous knockout condition. As for numerous other channelopathies, correlations of the nature of *SCN1A* variants (such as location in the channel, genetic mechanism, and associated impact on encoded protein function) with disease severity have been described (Zuberi et al., 2011). Our data demonstrate that primarily altered channel gating and accompanying changes in membrane properties can outweigh maintained physiological sodium peak current density and translate into pronounced functional impairment on neuronal level as frequently associated with DS.

## Methods

### Mutagenesis

The human *SCN1A* sequence cloned in the pCDM8 vector as described earlier (Hedrich et al., 2014) was corrected for two-point mutations, namely, Glu650Val and Ser1969Ala (Peters et al., 2016) by site-directed mutagenesis (Agilent Technologies, Santa Clara, CA, USA). The WT open reading frame included the canonical *SCN1A* adult isoform 2 (total length of 5997bp) equivalent to transcript NM_006920.5. The missense variant p.(Ala1783Val) was engineered into the human Na_V_1.1 channel by site-directed mutagenesis using PCR with Pfu polymerase (Promega; mutagenesis primers 5′ to 3′; F: ATG TAC ATC GTG GTC ATC CTG GAG AAC TTC AGT, R: AGG ATG ACC ACG ATG TAC ATG TTC ACC ACA). The introduced variant was verified, and further variants were excluded by sequencing the whole *SCN1A* cDNA. Plasmid purification was performed using *Escherichia coli* One Shot TOP10/P3 (Thermo Scientific, Waltham, MA, USA).

### tsA201 Cell Culture and Transfection

tsA201 cells were cultured in the Dulbecco's modified Eagle nutrient medium (Thermo Scientific, Waltham, MA, USA) supplemented with pyruvate, 10% v/v fetal bovine serum (Pan Biotech, Aidenbach, Germany), and 2 mM l-glutamine (Merck Millipore, Burlington, MA, USA) at 37°C in a 5% CO_2_ humidified atmosphere. For transfection, cells with a passage from P15 to P22 were used. Before transfection, 800,000–1,000,000 cells were split in 35-mm Petri dishes. Six hours later, cells were transfected by the following standard protocols: 4 μg of WT or mutant *SCN1A* cDNA, encoding the Na_V_1.1 channel-α-subunit, and 0.4 μg each of the human β_1_- and β_2_-subunits of voltage-gated Na_V_ channels, which had been previously modified to express either green fluorescent protein (pCLH-hb1-EGFP) or a CD8 marker (pCLH-hb2-CD8) to label cells expressing both subunits (Liao et al., 2010), were added to 250 μl of Opti-MEM (Gibco) supplemented with 7.5 μl of Mirus TransIT reagent (Mirus Bio LLC, Madison, WI, USA). After 20 min, the transfection mixture was added to the cells.

### Cortical Brain Slice Cultures

Coronal brain slices from conditional B6(Cg)-*Scn1a*^*tm*1.1*Dsf*^/J mice (JAX stock #026133) of either sex were obtained at P4-5. Mice were quickly decapitated, and cranium, brain stem, and hindbrain were removed. The forebrain was placed in ice-cold artificial cerebrospinal fluid (aCSF) bubbled with carbogen (95% O_2_/5% CO_2_). The aCSF contained the following [in mM]: 118 NaCl, 3 KCl, 1.5 CaCl_2_, 1 MgCl_2_, 25 NaHCO_3_, 1 NaH_2_PO_4_, 30 glucose, and pH 7.4 with an osmolarity of 310–320 mOsm/kg. Forebrains were glued with the cerebellar end facing downward on an agar block, and 350 μm thick slices were cut using a vibratome (Microm, H650 V) in ice-cold aCSF. Slices were placed in 36°C warm aCSF (bubbled with carbogen) for 10 min. Coronal slices containing the somatosensory cortex were transferred to Millicell cell culture inserts (Merck Millipore, Burlington, MA, USA) floating on the slice culture medium (Minimum Essential Medium Eagle with 20% horse serum, 1 mM l-glutamine, 0.00125% ascorbic acid, 0.001 mg/ml insulin, 1 mM CaCl_2_, 2 mM MgSO_4_, 1% penicillin/streptomycin, 13 mM glucose, pH 7.28, and osmolarity 320 mOsm/kg).

### Electrophysiological Recordings in tsA Cells and Slice Cultures

Whole-cell voltage-clamp recordings of tsA201 cells were performed 48 h after transfection from cells expressing all three sodium channel subunits indicated by an inward sodium current (α-subunit), anti-CD8 antibody-coated microbeads (Dynabeads M450, Dynal) (β_1_-subunit), and green fluorescence (β_2_-subunit). Cells were split in 35-mm Petri dishes 1 h prior to recordings, and each dish was used for up to 1 h after transfer to the patch-clamp setup. The extracellular bath solution contained the following [in mM]: 140 NaCl, 4 KCl, 1 MgCl_2_, 2 CaCl_2_, 5 HEPES, and 4 glucose. pH was adjusted with HCl to 7.4, and osmolarity was 300–305 mOsm/kg. Patch pipettes were pulled from borosilicate glass (Science Products GmbH) using a P97 Puller (Sutter Instruments), with resistances of 1.5–3.0 MΩ. Intracellular solutions for patch pipettes contained the following [in mM]: 5 NaCl, 2 MgCl_2_, 5 EGTA, 10 HEPES, 130 CsF, pH 7.4 (adjusted with CsOH), and osmolarity 290–295 mOsm/kg.

Whole-cell current-clamp recordings of excitatory pyramidal cells (PCs) and FS-INs in the somatosensory cortex of organotypic cortical slice cultures of conditional or WT animals were performed 7–14 days after adeno-associated viral transduction with 1 μl/slice of AAV8-hSyn-Cre-GFP (1 × 10^13^ VG/ml; SignaGen Laboratories, Frederick, MD, USA). Slices were positioned in a submerged-type recording chamber (Luigs & Neumann, Ratingen, Germany), continuously superfused with oxygenated recording aCSF, and maintained at a temperature of 33 ± 1°C. Transduced neurons were visualized using an Axioskop 2FS microscope (Carl Zeiss, Oberkochen, Germany). Patch pipettes had resistances of 2.5–5.5 MΩ. Intracellular solutions contained the following [in mM]: 140 K-gluconate, 1 CaCl_2_, 10 EGTA, 2 MgCl_2_, 4 Na_2_-ATP, 10 HEPES, 0.45% biocytin (Sigma-Aldrich, B4261), pH 7.2, and osmolarity of 300–310 mOsm/kg. PCs and FS-INs were used for current-clamp recordings. Cell identity was determined *via* morphological and electrophysiological properties. To block postsynaptic AMPA receptor-driven depolarization waves, 20 μM CNQX (Sigma-Aldrich, St. Louis, MO, USA) was added to the bath solution before recording.

### Immunohistological Stainings of Cortical Slice Cultures and Imaging

Following recordings, slices were fixed for 24 h in 4% paraformaldehyde (Morphisto GmbH, Offenbach am Main, Germany), washed in DPBS (Thermo Fisher Scientific, Waltham, MA, USA, Cat. 12559069), and blocked with 1% normal goat serum with 0.2% Triton X-100 for 2 h. AAV-positive cells were stained with rabbit anti-GFP IgG primary antibody (dilution 1:1,000, Invitrogen, Cat. A-11122) overnight at 4°C. Subsequently, slices were washed in DPBS and counterstained with goat anti-rabbit IgG secondary antibody Alexa Fluor-488 (dilution 1:200, Invitrogen, Cat. A-11008) and Streptavidin-Cy3 (dilution 1:100, Sigma, Cat. S6402) for 3 h at room temperature. After washing, slices were mounted with DAPI Fluoromount-G (Southern Biotech, Birmingham, AL, USA, Cat. 011-20). Immunohistological images of stained slices were acquired on a Zeiss Axiovert 200M microscope (widefield images) and a Leica SP8 microscope (confocal images).

### *In silico* Modeling of Cortical Neurons/Computational Modeling

Simulations were run using custom Python 3.7 software. A single-compartment conductance-based model with a cylindrical shape with length *L* and diameter *d* was used. The model is based on Pospischil et al. (2008) and consists of two sodium currents (*I*_*Na,wt*_ and *I*_*Na,mut*_), a delayed rectifier potassium current *I*_*K*_, a M-type potassium current *I*_*M*_, and a leak current *I*_*leak*_:


$$\begin{array}{lll} C_{m}\overset{\cdot }{V} & = & -I_{Na,wt}-I_{Na,mut}-I_{K}-I_{M}-I_{leak}+I_{input}\\ I_{Na,wt} & = & g_{Na,wt}m_{wt}^{3}h_{wt}s_{wt}\left[V-E_{Na}\right]\\ I_{Na,mut} & = & g_{Na,mut}m_{mut}^{3}h_{mut}s_{mut}\left[V-E_{Na}\right]\\ I_{K} & = & g_{K}n^{4}\left[V-E_{K}\right]\\ I_{M} & = & g_{M}p\left[V-E_{K}\right]\\ I_{leak} & = & g_{leak}\left[V-E_{leak}\right] \end{array}$$

with membrane capacitance $C_{m}=1\;\mu F/cm^{2}$, input current *I*_*input*_, maximal conductance *g*_*i*_, reversal potential *E*_*i*_, gating variables *m, h, n*, and *p* with dynamics


$$\begin{array}{l} {ẋ}_{i}=\alpha_{i}\left(V-shift_{i}\right)\left[1-x_{i}\right]-\beta_{i}\left(V-shift_{i}\right)x_{i} \end{array}$$

and the slow inactivating gating variable *s* with


$$\begin{array}{l} \tau_{s}ṡ=\frac{1}{1+\exp\left(-\left[V-V_{h}-shift_{s}\right]/k\right)}-s \end{array}$$

where ẋ_*i*_ is the derivative of the gating parameter with respect to time, α_*i*_(*V*) is the opening rate, and β_*i*_(*V*) is the closing rate of the respective gate *x*_*i*_. Steady-state curves and time constants are given by *x*_∞, *i*_(*V*) = α_*i*_(*V*)/[α_*i*_(*V*) + β_*i*_(*V*)] and τ_*i*_(*V*) = 1/[α_*i*_(*V*) + β_*i*_(*V*)]. The dynamics of the slow inactivating gating variable *s* followed


$$\begin{array}{l} \tau_{s}ṡ=\frac{1}{1+exp\left(\frac{-\left[V-V_{h}-shift_{s}\right]}{k}\right)}-s \end{array}$$

As suggested in the study by Pospischil et al. (2008), parameters for gating variables *m, h*, and *n* were taken from Traub and Miles (1991) and for gating variable *p* from Yamada (1989). We constructed the slow inactivation gate *s* to be similar to observed kinetics as suggested by Vilin and Ruben (2001). To simulate different neuron types, the conductivities of the ionic currents were adapted from the study by Pospischil et al. (2008). All parameters that differ from the study by Pospischil et al. (2008) are summarized in Table 1. Effects of variants were simulated with changes in the shifting parameter *shift*_*i*_ for the activation and slow inactivation sodium gates with *shift*_*i*_ = 0 *mv* unless stated otherwise.

**Table 1:** Simulation parameters for the cortical neuron models.

	**E_Na_ [mV]**	**E_K_ [mV]**	**E_L_ [mV]**	**g_K_ [**μ**S/cm^2^]**	**g_M_ [**μ**S/cm^2^]**	τ_max_, **_M_ [ms]**	**k_s_ [mV]**	**V_h, s_ [mV]**	**d,L [μm]**
IN	50	−90	−65	6.6	0.0485	934	−10	−60	60
PC				4.8	0.065	1123.5			68
**Varying parameters**									
		$\text{N}{\text{a}}_{\text{v}}1.1^{+/+}$	$\text{N}{\text{a}}_{\text{v}}1.1^{+/\text{A}1783\text{V}}$	**Activation shift**	**Slow inactivation shift**	**Slow inactivation time constant**	$\text{N}{\text{a}}_{\text{v}}1.1^{+/-}$	$\text{N}{\text{a}}_{\text{v}}1.1^{+/+}+{\text{I}}_{\text{L}}$	$\text{N}{\text{a}}_{\text{v}}1.1^{+/\text{A}1783\text{V}}+{\text{I}}_{\text{L}}$
IN	$g_{Na,wt}\;\left[\mu S{/cm}^{2}\right]$	60.7	60.7	60.7	60.7	60.7	60.7	60.7	60.7
	$g_{Na,mut}\;\left[\mu S/cm^{2}\right]$	23.3	23.3	23.3	23.3	23.3	0.0	23.3	23.3
	$g_{L}\;\left[\mu S/cm^{2}\right]$	0.274	0.274	0.274	0.274	0.274	0.274	0.365	0.365
PC	$g_{Na,wt}\;\left[\mu S/cm^{2}\right]$	45.8	45.8	45.8	45.8	45.8	45.8	45.8	45.8
	$g_{Na,mut}\;\left[\mu S/cm^{2}\right]$	4.2	4.2	4.2	4.2	4.2	0.0	4.2	4.2
	$g_{L}\;\left[\mu S/cm^{2}\right]$	0.105	0.105	0.105	0.105	0.105	0.105	0.124	0.124
*shift*_*m*_ [*mV*]		0.0	10.0	10.0	0.0	0.0	0.0	0.0	10.0
*shift*_*s*_ [*mV*]		0.0	−15.0	0.0	−15	0.0	0.0	0.0	−15.0
τ_*s*_[s]		30.0	3.0	30.0	30.0	3.0	30.0	30.0	3.0

To simulate the effects of heterozygous variants in *SCN1A* on the firing behavior of PCs and interneurons, different ratios of Na_V_1.1 and Na_V_1.6 expression were modeled in the respective neuron types with the assumption of equal gating properties in both WT sodium channels (*g*_*Na,wt*_ = *g*_*NaV*1.1_/2 + *g*_*NaV*1.6_) and potentially altered Na_V_1.1 channels (*g*_*Na,mut*_ = *g*_*NaV*1.1_/2). The ratio of assumed sodium conductance was *g*_*NaV*1.6_ = 5*g*_*NaV*1.1_ for pyramidal neurons and *g*_*NaV*1.6_ = 0.8*g*_*NaV*1.1_ for FS-INs based on expression data from cortical mouse neurons (Yao et al., 2020). To investigate how each observed change in gating parameters contributes to the observed change in firing behavior, we constructed several variant models, one combined variant model with all parameter changes, and for each adapted parameter, one model with only one respective change.

The excitability of these models was computed with the *f*−*I* curve. We simulated square pulse stimuli between 0 and 500 pA with increments of 5 pA. Action potential (AP) frequency of each step was computed by dividing the number of measured spikes, reduced by 1, with the time difference between the first and the last spike:


$$\begin{array}{l} f\left(I\right)=\left[\#spikes-1\right]/\left[t_{last\;spike}-\;t_{first\;spike}\right] \end{array}$$

### Statistical Analysis

All data are displayed as mean ± SEM. Statistical testing was performed *via* unpaired, two-tailed *t*-test for normally distributed data and Mann–Whitney *U* test for non-normally distributed data and *n* indicates the number of cells. Data normality was tested by using the D'Agostino and Pearson normality test. Mean-, *n-* and *p*-values are shown in Supplementary Tables 1–3.

All data were analyzed using pClamp 10.703 (Molecular Devices, LLC, San Jose, CA, USA), Microsoft Excel (Microsoft Corporation, Redmond, WA, USA). Statistics were performed using GraphPad 7 (GraphPad Prism, San Diego, CA, USA).

## Results

### Biophysical Characterization of *SCN1A^*A*1783*V*^* Channel Function in tsA201 Cells Reveals LOF With Maintained Peak Sodium Currents

**Figure 1:**
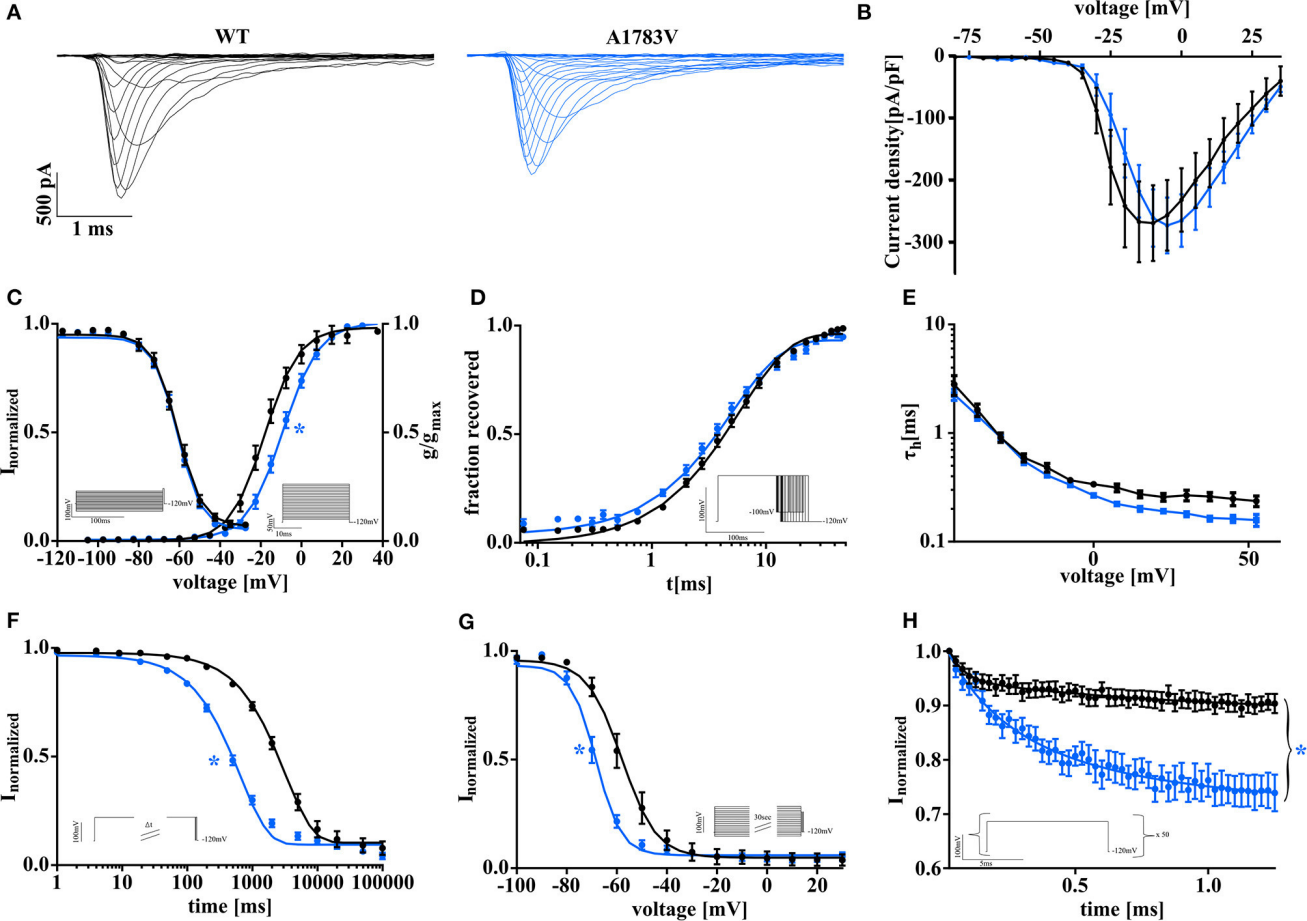
Biophysical properties of *SCN1A* wild type and mutant Na_V_1.1^A1783V^ channels recorded in tsA201 cells display a clear loss of function. **(A)** Representative current traces were recorded from tsA201 cells transfected with Na_V_1.1^WT^ (black traces) or Na_V_1.1^A1783V^ (blue traces). **(B)** Peak Na^+^ currents normalized by cell capacitance were plotted vs. command voltage and revealed no changes between wild-type (WT) and mutant Na_V_1.1^A1783V^ channels. (**C**, right) Voltage-dependence of steady-state activation curves represented as normalized conductance fit by a Boltzmann function to the data points (lines). (**C**, left curve) Voltage-dependent state of fast inactivation fit by a Boltzmann function to the data points (lines). **(D)** Time course of recovery from fast inactivation at −100 mV. **(E)** Voltage-dependence of the time constant of fast inactivation τ_*h*_ analyzed by a one exponential fit to inactivating phase of the Na^+^ currents. **(G)** Entry into slow inactivation is represented as a first-order exponential fit to the data points. **(H)** Voltage-dependent steady-state of slow inactivation fit by a Boltzmann function as in **(C)**. **(F)** Na-current use dependence evoked by 50 pulses at a frequency of 40 Hz as normalized peak currents plotted against the time and fit by a second-order exponential function. The number of recorded cells, exact values of statistically analyzed parameters, and *p*-values are listed in Supplementary Table 1.

Wild-type or mutant Na_V_1.1 channels were transfected into tsA201 cells, and whole-cell patch recordings were performed. Representative raw current traces are shown in Figure 1A. While peak current density was not changed for mutant channels in comparison to WT (Figure 1B), activation and inactivation properties were markedly altered. We found a significant right shift of the half-maximal conductance indicating that mutant channels open and reach their maximal activation at more depolarized potentials (Figure 1C). Fast inactivation was unaffected as revealed by comparable voltage-dependence of steady-state fast inactivation (Figure 1C), time constant τ (Figure 1E), and recovery from fast inactivation (Figure 1D) for WT and Na_V_1.1^A1783V^ channels. In contrast, entry into slow inactivation was accelerated (Figure 1F), and voltage-dependence was shifted to more hyperpolarized potentials (Figure 1G). Overall, Na_V_1.1^A1783V^ channel LOF became very obvious on rapid successive depolarizing stimulations at 40 Hz revealing a markedly increased use-dependence with consecutively pronounced “rundown” of the sodium current (Figure 1H).

### *In silico* Modeling Reveals Pronounced Interneuron Firing Impairment by Na_V_1.1^A1783V^

**Figure 2:**
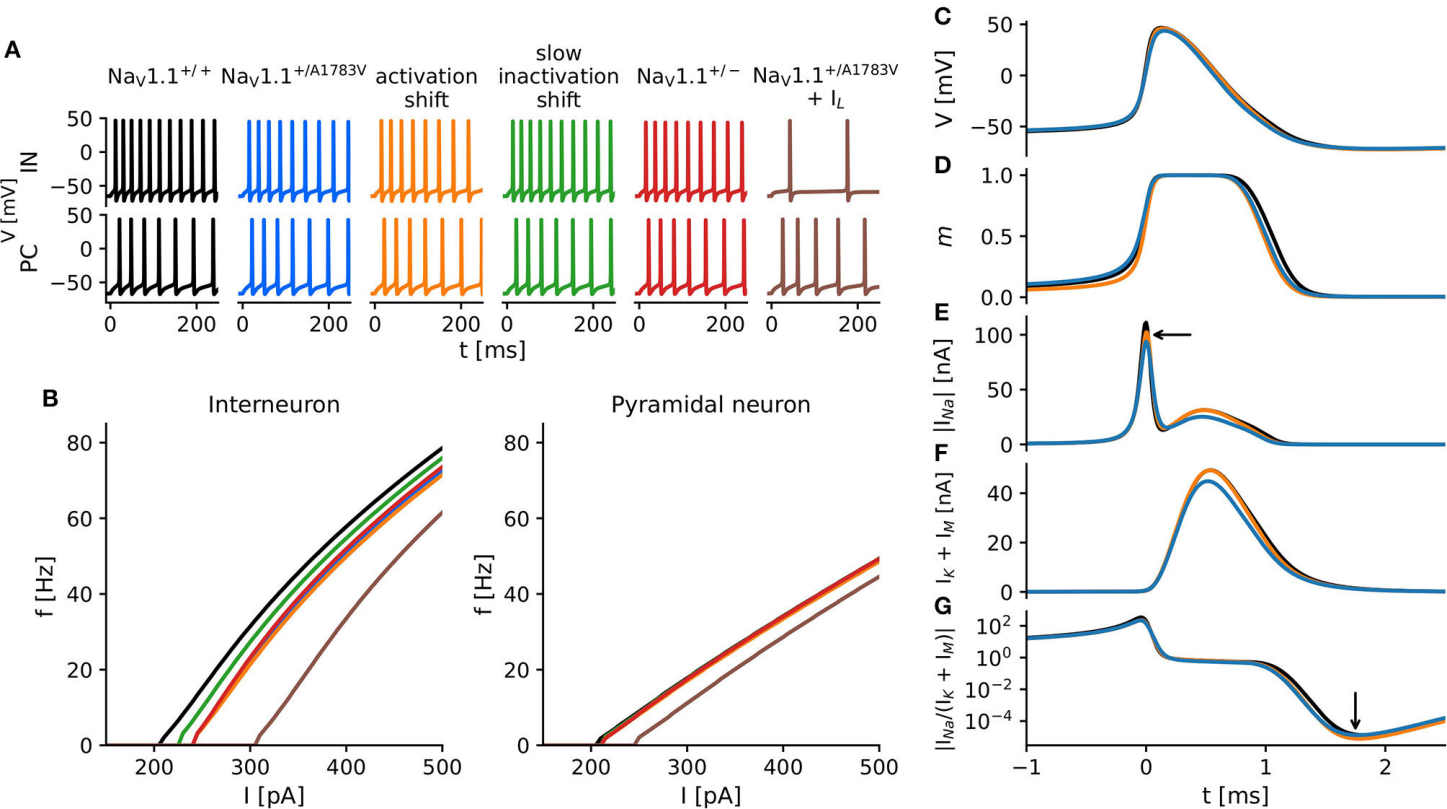
*In silico* modeling of neuron excitability. **(A)** Action potentials of simulated cortical fast-spiking interneurons (upper traces) and cortical pyramidal cells (PCs) (lower traces) in response to square pulse current injections of 320 pA with various modifications of Na_V_1.1 current kinetics based on findings in tsA recordings. Colors indicate different model conditions: WT (black), the heterozygous shift of the voltage-dependence of steady-state activation (orange), the heterozygous shift of the voltage-dependence of the steady-state slow inactivation (green), the combination of both changes (blue), a heterozygous protein knockout (red), and a combination of respective changes in sodium current and a decreased input resistance of the cell (brown). The exact parameters for all simulated conditions are summarized in Table 1. **(B)** *f*−*I* curves of interneurons (left) and PCs (right) constructed from simulation studies displayed in **(A)**. **(C–G)** Time courses of model variables during the first action potential after a 300 pA current injection, *t* = 0 indicates the peak of the action potential. **(C)** Membrane voltage; **(D)** activation gate *m*—ratio of activated sodium channels; **(E)** absolute *I*_Na_; **(F)** *I*_K_; and **(G)** the absolute ratio of *I*_Na_ to *I*_K_.

Full haploinsufficiency is considered as the genetic mechanism underlying the majority of *SCN1A* Dravet variants. About half of the described variants lead to protein truncation with reduced protein expression in affected neurons and likely markedly reduced conductance (Marini et al., 2011). Our recordings revealed clear biophysical LOF changes of Na_V_1.1^A1783V^ compared with WT, however no reduction of peak current density. Then, we asked how these combined functional alterations translate to the neuronal level and how they compare to the heterozygous knockout condition. To address these questions, we built a single-compartment conductance-based model with the combined biophysical alterations reflecting intrinsic and firing properties of either cortical FS-INs or excitatory PCs neurons. Differential Na_V_1.1–Na_V_1.6 expression ratios of FS-IN and PCs and either the WT (Na_V_1.1^+/+)^, the mutant (Na_V_1.1^+/A1783V^), or the full haploinsufficiency (Na_V_1.1^+/−^) condition were implemented. The parameters for the different model types are summarized in Table 1. Additionally, we simulated altered voltage-dependence of activation as well as altered voltage-dependence and kinetics of slow inactivation separately to dissect their joint or exclusive impact on neuron action potential firing in comparison to the WT and the full haploinsufficiency condition (Figure 2A).

In the interneuron model, solely shifting the activation curve to more depolarized membrane potentials caused the most severe reductions of the firing rates in comparison to the WT, closely followed by the Na_V_1.1^+/A1783V^ and Na_V_1.1^+/−^ conditions. Shifting the slow inactivation curve to more hyperpolarized potentials reduced firing rates to a lesser extent (Figure 2B, left). Although the faster slow inactivation model was the only simulation yielding an accelerated rundown of sodium current amplitudes as had been observed in tsA201 cells expressing Na_V_1.1^A1783V^ (Supplementary Figure 1C), it showed almost no impact on firing rates in comparison to WT (Supplementary Figures 1A,B). The firing properties of all pyramidal models remained largely unaffected (Figure 2B, right; Supplementary Figure 1B).

To better understand why shifting the activation curve for half of the Na_V_1.1. channels (to mimic the heterozygous patient situation) had a stronger effect on neuronal firing than the simulation of the heterozygous protein knockout, we analyzed the current dynamics of the first action potential in more detail (Figures 2C–G). Although on a first glance, the action potential waveforms seemed to be rather similar (Figure 2C), the sodium activation gate (Figure 2D) of the model with a shifted activation curve (orange) opened later and closed earlier compared with the WT (black) and the Na_V_1.1^+/−^ models (blue). The resulting sodium current was reduced during AP initiation and rising phase of the action potential compared to the WT simulation but was similar during the falling phase of the action potential (AP). Only at the very end of the AP, the sodium current appeared again slightly reduced. In contrast, the Na_V_1.1^+/−^ model depicted a generally reduced sodium current (Figure 2E), which was followed by a pronounced reduction of the delayed rectifier potassium current. This effect on the potassium current was almost absent when modeling a right-shifted activation curve of Na_V_1.1. (Figure 2F). In combination with the observed effects on the sodium current, the right-shifted activation curve model showed a greater reduction of the sodium-to-potassium current ratio in the final phase of the afterhyperpolarization than the Na_V_1.1^+/−^ model (Figure 2G). This increased after-hyperpolarization lead then to a delay of the next action potential.

### Na_V_1.1^A1783V^ LOF Is Restricted to Inhibitory Neurons in Cortical Mouse Brain Slice Cultures

**Figure 3:**
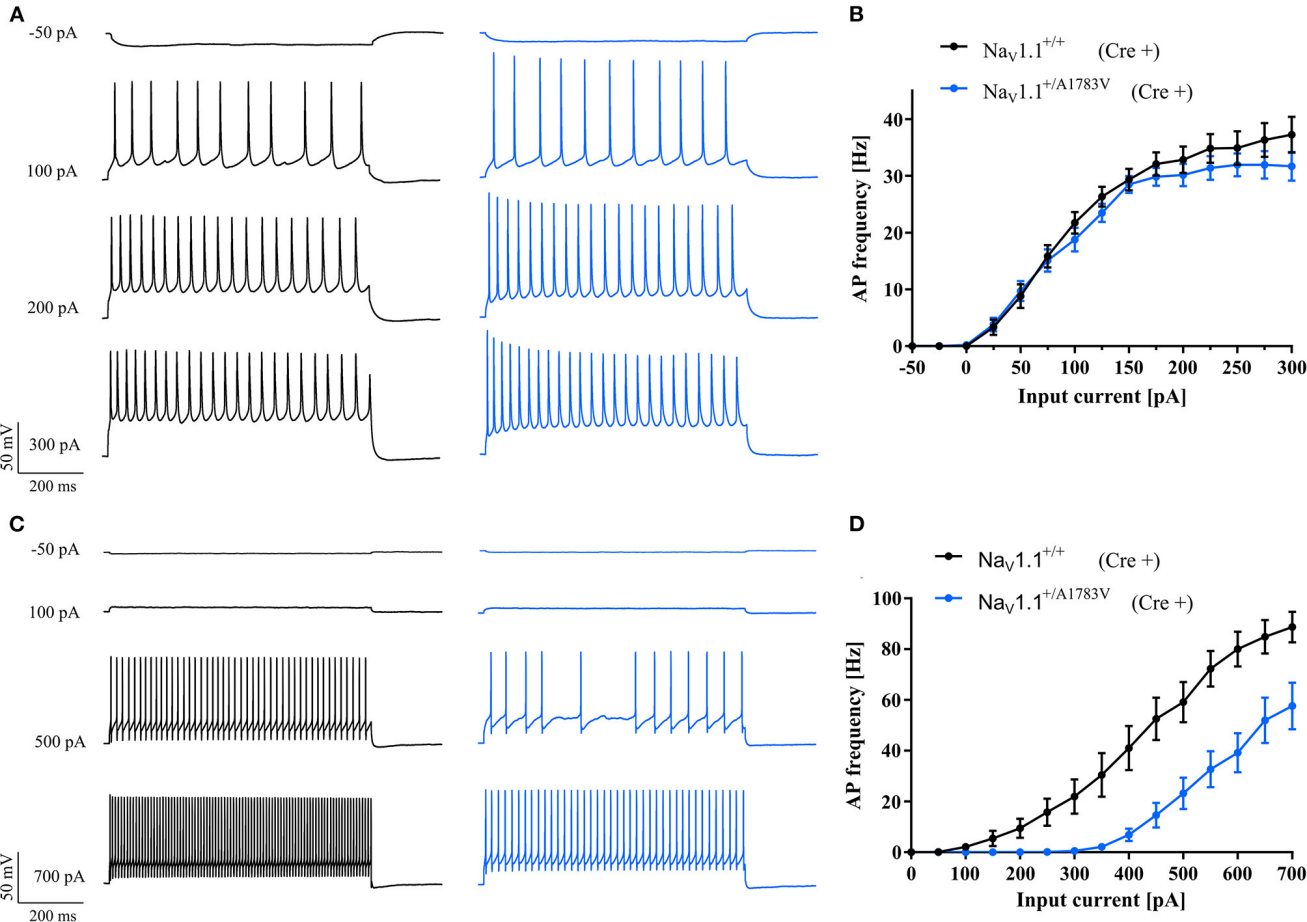
*In vitro* studies of neuronal excitability. **(A)** Representative AP trains in response to 800 ms step current injections from cortical PCs and **(C)** fast-spiking interneurons recorded in brain slice cultures of WT (black traces) and heterozygous Na_V_1.1^+/A1783V^ animals (blue traces). **(B)** *f*−*I* curves of PCs from floxed heterozygous mice showed the same frequency of action potential firing while **(D)** fast-spiking inhibitory neurons showed higher and maintained lower AP frequencies compared to cells of WT animals. A number of recorded cells, exact values of statistically analyzed parameters, and *p*-values are listed in Supplementary Tables 2, 3.

To confirm the simulated effects of Na_V_1.1^A1783V^ on neuronal firing, we performed whole-cell patch-clamp recordings in cortical brain slice cultures derived from P4-5 heterozygous B6(Cg)-*Scn1a*^*tm*1.1*Dsf*^/J mice and WT littermates. Cultures were virally transduced at day 1 *in vitro* with AAV8 Cre-recombinase-GFP under the human synapsin promoter to induce pan-neuronal recombination and subsequent expression of the Na_V_1.1^+/A1783V^ variant. Since *Scn1a* is upregulated in the postnatal period only from P11 onward (Cheah et al., 2013), this early expression of Cre-recombinase allowed for recombination of transduced neurons carrying the floxed *Scn1a*^*A*1783*V*^ allele before endogenous upregulation of the *Scn1a* gene. This approach ensured activation of the variant following the endogenous expression time course of the Na_V_1.1 channel. Notably, 7–14 days after transduction, GFP+ neurons were patched (Supplementary Figure 2). While the firing of PCs was not altered (Figures 3A,B), we found a LOF in FS-INs (Figures 3C,D). Na_V_1.1^+/A1783V^ expressing FS-INs displayed a significantly increased rheobase, reduced maximum firing frequency, and reduced input resistance compared to WT controls (Supplementary Table 2). Similarly, we could detect a trend for a reduction of the input resistance of PCs (Supplementary Table 3). However, the firing properties of PCs were not significantly altered. AP threshold, AP amplitude, AP rise time, AP half-width, and afterhyperpolarization amplitude within evoked AP trains were indistinguishable between neurons (FS-INs and PCs) recorded from slices of WT and mutant animals. We applied the observed changes in input resistance to the *in silico* model (Figure 2, brown). These simulations combining changed gating and membrane properties predicted an even stronger LOF in FS-INs while only tentatively affecting the firing of simulated PCs.

## Discussion

Full haploinsufficiency is considered the leading genetic cause of DS, resulting in impaired excitability predominantly of interneurons (Catterall et al., 2008). In this study, we functionally characterized the human recurrent *SCN1A*^*A*1783*V*^ DS variant. Although reduced-sodium peak current density is a common feature associated with DS, our analysis revealed altered activation and slow inactivation properties, albeit with preserved sodium peak current density. However, an enhanced use-dependent rundown of Na^+^ current was observed on repetitive stimulations. Although an interference of the variant (localized at the end of segment 6 in domain 4) with local determinants of fast inactivation would have been conceivable (Ulbricht, 2005), fast inactivation was found to be unchanged. Since fast inactivation was unaffected, use-dependence can only be attributed to slow inactivation characteristics of the variant. In fact, this could be confirmed by *in silico* modeling of Na^+^ current with physiological or accelerated slow inactivation kinetics (Supplementary Figure 1C). Interestingly, modeling of neuronal excitability predicted a fairly strong overall interneuron AP firing deficit for Na_V_1.1^+/A1783V^ at comparable levels to the simulated full haploinsufficiency condition (Na_V_1.1^+/−^). Since our tsA201 cell recordings had revealed a ~25% sodium current “rundown” on 40 Hz stimulation, we speculated that altered slow inactivation and associated enhanced use-dependence might largely account for the predicted interneuron firing deficit. Importantly, the model correctly predicted enhanced use-dependence of Na_V_1.1^*A*1783*V*^ currents and confirmed accelerated slow inactivation as the only underlying mechanism (Supplementary Figure 1C). However, when simulating *f*−*I* curves for FS-INs with different time constants of slow inactivation, an acceleration of τ only had a marginal effect on neuronal firing (Supplementary Figure 1A) and shifted voltage-dependence of fast activation was predicted as the main driver of the impaired interneuron firing. Another observation from our recordings in cortical brain slice cultures was a significant reduction of input resistance in neurons expressing p.A1783V. These changes in passive membrane properties might also contribute to the observed IN LOF and are consistent with recordings of hippocampal INs in brain slices of mice harboring p.A1783V (Almog et al., 2021). However, the underlying mechanisms of altered input resistance remain still to be addressed in a follow-up study. At this point, it is hard to project how the pA1783V variant would directly account for this change, and secondary cellular changes affecting membrane leak properties may have to be considered.

In our simulations, the comparison of Na_V_1.1^+/A1783V^ with the simulated Na_V_1.1^+/−^ condition revealed a similar action potential deficit. Although surprising at first, these findings could at least partially be explained when putting the activation properties and relative levels of conducted currents of Na_V_1.1^+/A1783V^ and Na_V_1.1^+/−^ in context with the different phases of the action potential. Conductance-based modeling suggested a reduced number of sodium channels available to participate in action potential initiation due to the shifted activation curve and subsequently delayed opening of the channels. This effect was qualitatively similar to Na_V_1.1^+/−^ (with only a limited number of available channels due to the simulated heterozygous knockout condition) in this early phase of an action potential and was predicted to account for most of the overall LOF. However, in the falling phase of the action potential, Na_V_1.1^+/A1783V^ yielded larger sodium currents than Na_V_1.1^+/−^ and accordingly recruited more potassium channels. Our simulations predicted that the induced potassium current outweighed the conducted sodium current in the final hyperpolarizing phase in neurons of the heterozygous Na_V_1.1^+/A1783V^ condition. This relative surplus of K^+^ current led to a slightly pronounced afterhyperpolarization (0.5–1.0 mV) and a prolonged time window until the next spike could occur, altogether increasing the LOF effect. This predicted change on afterhyperpolarization was reflected as a nonsignificant tendency in our brain slice culture recordings (Supplementary Table 2). Consistent with our data, pronounced shifts of voltage-dependence of activation have also been described for other LOF mutations associated with epilepsy, such as *SCN1A*^I1656M^ (Lossin et al., 2003), *SCN1A*^D249E^ (Kluckova et al., 2020), and *SCN1A*^R859C^ (Barela et al., 2006). Similar to S*CN1A*^A1783V^, these variants also feature unaltered current density in comparison to the WT.

Na_V_1.1 is predominantly expressed in interneurons (Yao et al., 2020), while PCs mainly rely on Na_V_1.6 for action potential initiation. Therefore, a leading dysfunction of interneurons was expected and was indeed reflected *in silico*. These data were confirmed in cortical brain slice cultures derived from conditional *Scn1a*^+/A1783V^ mice. Our findings are consistent with data on hippocampal interneurons expressing Na_V_1.1 ^+/A1783V^, which also displayed a shift in rheobase and a firing deficit at higher current injections (Almog et al., 2021).

## Conclusion

*SCN1A*^A1783V^ results in altered voltage-dependence of activation and slow inactivation while maintaining sodium peak current density. Interneuron excitability was simulated *in silico* by using a Hodgkin-Huxley one-compartment model suggesting largely similar firing impairment for Na_V_1.1^+/A1783V^ in comparison to Na_V_1.1^+/−^ resembling full haploinsufficiency. Impaired Na_V_1.1^A1783V^ voltage-dependent activation was suggested as the main primary mechanism underlying interneuron dysfunction.

## Data Availability Statement

The original contributions presented in the study are included in the article/Supplementary Material, further inquiries can be directed to the corresponding author/s.

## Ethics Statement

The animal study was reviewed and approved by local Animal Care and Use Committee (Regierungspraesidium Tuebingen, Tuebingen, Germany).

## Author Contributions

NL did the molecular biology, cultured brain slices, and performed current-clamp recordings in brain slice cultures. NL and EP performed voltage-clamp recordings in tsA201 cells. LS and JB developed *in silico* model. NL and LS did the data analysis. NL, LS, JB, HK, and TW wrote the manuscript. NL, LS, EP, JB, UBSH, HL, HK, and TW did the proof reading and study design. All authors contributed to the article and approved the submitted version.

## Funding

This study was supported by the German Research Foundation (DFG/FNR INTER research unit FOR2715 grants Ko4877/3-1, Le1030/15-1, He8155/1-1). TW was supported by an intramural Clinician Scientist Fellowship granted by the Faculty of Medicine, University of Tübingen (419-0-0). We acknowledge support by Open Access Publishing Fund of University of Tübingen.

## Conflict of Interest

The authors declare that the research was conducted in the absence of any commercial or financial relationships that could be construed as a potential conflict of interest.

## Publisher's Note

All claims expressed in this article are solely those of the authors and do not necessarily represent those of their affiliated organizations, or those of the publisher, the editors and the reviewers. Any product that may be evaluated in this article, or claim that may be made by its manufacturer, is not guaranteed or endorsed by the publisher.
