# Supplementary material for: Dravet Variant SCN1AA1783V Impairs Interneuron Firing Predominantly by Altered Channel Activation
Source: Front Cell Neurosci. 2021 Oct 28;15:754530. doi: 10.3389/fncel.2021.754530 (PMC8581729; doi:10.3389/fncel.2021.754530)
Supplement: Supplementary file 1 [file Data_Sheet_1.pdf]

Supplementary Material

Supplementary materials and methods

Whole cell voltage clamp data acquisition in tsA201 cells

Patched TsA201 cells were compensated manually for pipette capacitance, cell capacitance and series resistance. Cells with a voltage error lager than 4 mV, a peak current smaller than 750 pA or a series resistance larger than 20 MΩ were excluded from analysis. Series resistance typically ranged between 5 and 15 MΩ. Series resistance compensation was applied up to 90%.

Signals were amplified with an Axopatch 200B (Molecular Devices) amplifier, low-pass filtered with a Bessel filter at 10 kHz, sampled at 20 kHz, digitized (DigiData 1440, Molecular Devices) and recorded with pClamp 10.7 software (Molecular devices). During recordings cells were held at -120 mV. Leak subtraction was performed before each sweep by four sub-pulses with opposite polarity of the applied stimulus (-P4).

For measuring channel activation, a 25 ms long square pulse ranging from -105 to 45 mV in 7.5 mV increments was applied. Activation kinetics were analyzed from the elicited peak current of each sweep.

Time constant of fast inactivation was also deduced from the activation protocol. τ_h was examined for every sweep yielding an inward sodium current.

Voltage-dependence of fast inactivation was determined from applying the following stimulus protocol: A conditioning pulse was applied at various voltages ranging from -170 to -27.5 mV in 7.5mV increments for 300 ms. In succession, the remaining sodium current was elicited via a 3 ms long test pulse at -20 mV.

Time dependence of recovery from fast inactivation was recorded by depolarizing the cell and thereby inactivating sodium channels to -20 mV for 100 ms followed by a recovery period at -100 mV for various time periods ranging from 3 to 1900 ms. After recovery, a 7.5 ms test pulse at -20 mV was used to check for the recovered fraction of the sodium current.

Use dependence of $Na_V1.1$ was examined by applying a total of 50 voltage step pulses to -20 mV with a frequency of 40 Hz to simulate channel activation during an action potential train. Data was normalized on the current amplitude of the first resulting current peak.

Time course and voltage dependence of sodium channel slow inactivation were determined from two different stimulus protocols: Data for calculating the time constant of slow inactivation τ_{entry} was acquired by first applying a conditioning pulse at 0 mV with a duration ranging from 1 to 50000 ms followed by 5 ms test pulse at -20 mV to check the fraction of non-inactivated channels. For voltage dependence of slow inactivation, the duration of the conditioning pulse was locked instead to 30 s with varying voltage steps from -140 to +30 mV. The remaining fraction of non-inactivated channels was again checked by a test pulse to -20 mV for 5 ms directly after the conditioning pulse (Alekov et al., 2001).

Whole cell voltage clamp data analysis

Currents for activation and inactivation properties of $Na_V1.1$ sodium channels expressed in tsA201 cells were recorded as described above. The sodium reversal potential was calculated for each cell via

$$g_{Na}(V) = \frac{I}{[V - E_{Na}]}$$

with sodium peak current *I*, command voltage *V*, conductance g_{Na} and reversal potential E_{Na}. Conductance was determined by plotting the observed peak Na^+-current and fitting by

$$g_{Na}\ (V) = \frac{g_{max}[V - E_{Na}]}{1 + exp([V - V_{1/2}]/k_V)}$$

with maximal conductance g_{max}, slope factor k_V, half maximal activation $V_{1/2}$. Steady-state fast and slow inactivation were fit to the Boltzmann equation:

$$I_{Na}(V) = \frac{I_{max}}{[1 + exp([V - V_{1/2}]/k)] + c}$$

with maximal evoked sodium current I_{max} and *c* being an additional constant. To determine the time constant of fast inactivation τ_h, a first-order exponential function was fit inactivating component of the sodium current:

$$f(t) = Aexp\left(-\frac{t}{\tau_h}\right) + c$$

with maximal current *A*, time *t* and time constant of fast inactivation τ_h.

Recovery from fast inactivation was analyzed by fitting using a 1-exponential equation:

$$I_{Na}(t)\ =\ A[1 - exp\left(-\frac{t}{\tau_{rec}}\right)] + c$$

with maximally recovered current *A*, initial time delay t_0, time constant of recovery from fast inactivation τ_{rec}.

Entry into slow inactivation was fit by

$$I(t) =\ Aexp\left(-\frac{t}{\tau_{entry}}\right) +\ c$$

with the remaining peak current after various durations of depolarizing pre-pulse *I(t)*, maximal amplitude of peak current *A*, the duration of pre-pulse *t* and time constant of slow inactivation τ_{entry}.

Use dependence was fit by a second-order exponential equation with

$$I(t) = A_1 exp\left(-\frac{t}{\tau_1}\right) + A_2 exp\left(-\frac{t}{\tau_2}\right) + c$$

Whole cell current clamp data acquisition in cortical mouse brain slice culture

Patched neurons were compensated manually for pipette capacitance, cell capacitance and series resistance 5 min after rupturing the seal in voltage clamp. Cells with a leak current lager than -200 pA, an unstable series resistance, shifts in resting membrane potential or a series resistance larger than 20 MΩ were excluded from analysis. Series resistance correction was applied up to 85%.

Signals were amplified with an Axopatch 200B (Molecular Devices) amplifier, low-pass filtered with a Bessel filter at 10 kHz, sampled at 100 kHz, digitized (DigiData 1440, Molecular Devices) and recorded with pClamp 10.7 software (Molecular devices). Recordings of action potential trains were corrected for a liquid junction potential of 15 mV and clamped to -70 mV.

Current clamp protocols in slice cultures were structured as follows: Voltage traces to analyze input resistance were acquired by 500 ms long square pulse stimuli ranging from -10 to -110 pA with -10 pA increments. Action potential trains were evoked by 800 ms long square current injections (PCs: -50 to +300 pA in 25 pA steps; FS-INs: 0 to +700 pA in 50 pA steps) followed by a 5 s inter-sweep interval before the following current injection.

Whole cell current clamp data analysis

The resting membrane potential was determined as the mean membrane potential recorded over a duration of 2 minutes after compensation. The input resistance was determined as the slope of a linear regression fit to corresponding steady-state voltage responses of the last 300 ms of current injection plotted versus current injections.

Neuronal excitability was analyzed by plotting the mean action potential frequency over a 800 ms-long square current injection against the input current (f-I-curves). Only events with a voltage peak amplitude surpassing 0 mV were regarded as action potentials. Active properties of single action potentials were analyzed from the second action potential in a train at a step current injection of 600 pA for FS-INs and at 100 pA for PCs. The threshold of an action potential was determined as the voltage at which the first derivative *dV/dt* reached 20 mV/s. Action potential amplitude and rise time were measured from the threshold to the peak and the half-width was determined at 50% of the peak amplitude as the duration between the corresponding potentials of the rising and falling phases. Action potential afterhyperpolarization amplitude was calculated as the negative voltage amplitude from AP-threshold to the point the first derivative reached 0 mV/s in the falling phase of the AP.

Supplementary figures and tables

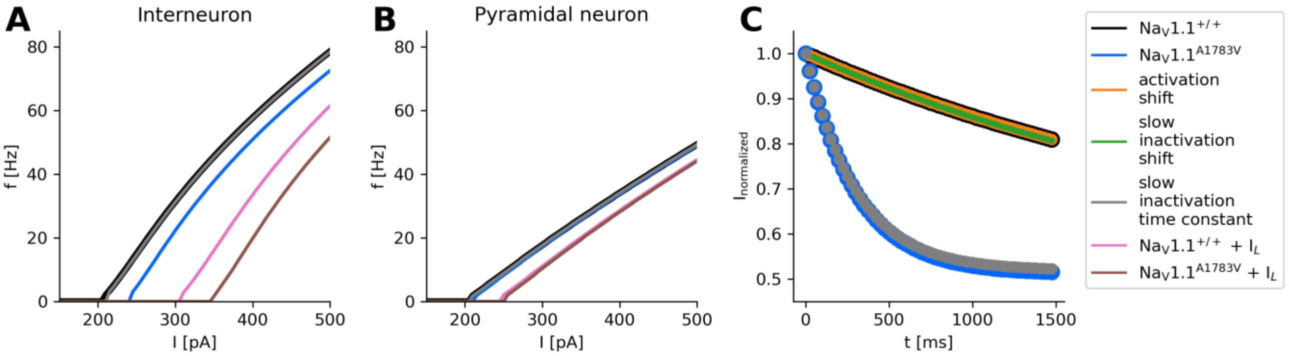

Supplementary Figure 1. Simulation study of accelerating the time constant of slow inactivation τ and sodium channel use dependence kinetics. F-I curves of modelled heterozygous cortical (**A**) interneurons and (**B**) pyramidal cells after acceleration of time constant of slow inactivation *τ* (gray) in comparison to WT (black) and mutant $Na_V1.1^{A1783V}$ (blue) based on findings in tsA cell recordings. Influence of changes in input resistance are shown alone (pink) and in combination with mutant $Na_V1.1^{A1783V}$ (brown). (**C**) Simulated use dependence of sodium current in a cell model solely expressing homozygous $Na_V1.1$ at 40 Hz after applying observed single parameter changes recorded in tsA201 cells expressing p.(Ala1783Val): changes of activation (orange), slow inactivation (green), time constant *τ* of slow inactivation (grey) and a combination of all three changed parameters reflecting $Na_V1.1^{A1783V}$ (blue) in comparison to WT (black).

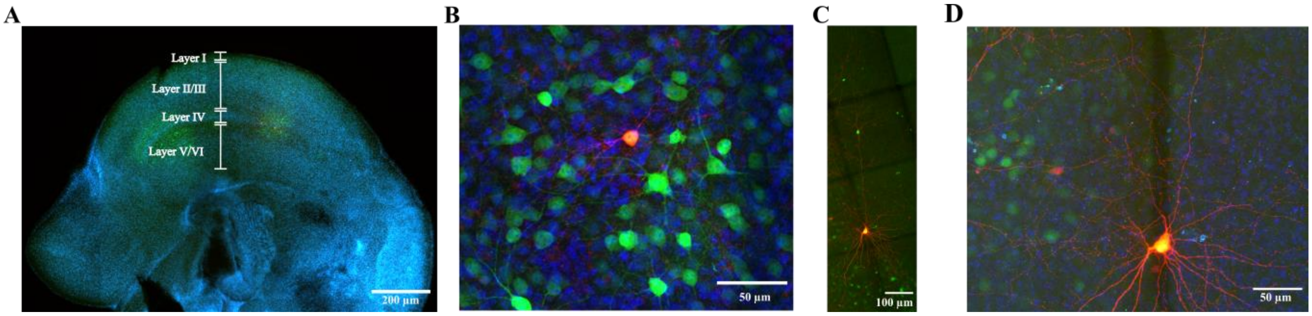

Supplementary Figure 2. Immunostainings of patched neurons in murine slice culture (**A**) Representative structure of a coronal prefrontal brain slice indicating the cortical layers after transduction with AAV8-hSyn-Cre-GFP (green fluorescence). Representative fast spiking interneurons (**B**) were recorded and filled with biocytin (0,45%) in cortical layer IV/V, pyramidal cells (**C, D**) in cortical layers V/VI and counterstained with Streptavidin-Cy3 (red fluorescence). Cortical layers were determined by neuronal density during patch-clamp recordings and post-hoc by Dapi staining (blue fluorescence).

Supplementary Table 1: Biophysical properties of $Na_V1.1$ wildtype and mutant $Na_V1.1^{A1783V}$ channels recorded in tsA201 cells.

	**Wildtype**	*n*	**A1783V**	*n*	*p*
**Current density [pA/pF]**	-282.2 ± 64.4	13	-284.1 ± 48.57	24	0.6043
**Steady-state of activation**					
$V_{1/2}$ [mV]	-16.72 ± 2.26	13	-9.17 ± 1.49	24	0.0066
k_V	-7.794 ± 1.02	13	-7.61 ± 0.31	24	0.3695
**Fast inactivation**					
$V_{1/2}$ [mV]	-61.31 ± 1.38	13	-61.1 ± 0.80	22	0.8863
k	5.54 ± 0.36	13	5.63 ± 0.37	22	0.6737
τ_{rec} at -100 mV [ms]	6.17 ± 0.57	13	5.30 ± 0.45	14	0.24
τ_h at 0 mV [ms]	0.34 ± 0.02	13	0.26 ± 0.02	24	0.0008
**Slow inactivation**					
$V_{1/2}$ [mV]	-57.93 ± 2.28	9	-68.44 ± 1.23	10	0.0006
k	6.07 ± 0.78	9	4.64 ± 0.31	10	0.0435
τ_{entry} [ms]	3321 ± 220.4	12	669.1 ± 38.12	16	< 0.0001
**Use dependence (40Hz)**					
%-I_{Na} at 50th pulse	0.903 ± 0.017	13	0.739 ± 0.034	12	0.0002

Supplementary Table 2: Electrophysiological properties of cortical fast spiking interneurons recorded in organotypic slice cultures from wildtype and floxed heterozygous *Scn1a*$^{+/A1783V}$ mice transduced with AAV8-Syn-Cre-GFP.

	**Wildtype**	*n*	**A1783V**	*n*	*p*
Resting membrane potential [mV]	-69.47 ± 0.72	16	-70.58 ± 1.03	16	0.390
Input resistance [MΩ]	107.8 ± 8.71	16	73.22 ± 8.87	16	0.003
Rheobase [pA]	209.4 ± 39.52	16	360.9 ± 40.05	16	0.012
Maximum firing frequency [Hz]	88.67 ± 6.00	16	57.66 ± 9.2	16	0.008
AP threshold [mV]	-25.62 ± 3.22	13	-30.3 ± 2.64	14	0.269
AP amplitude [mV]	49.65 ± 3.11	13	47.86 ± 2.74	14	0.668
AP rise time [ms]	0.75 ± 0.03	13	0.65 ± 0.04	14	0.092
AP half width [ms]	0.92 ± 0.05	13	0.78 ± 0.06	14	0.117
AP afterhyperpolarization amplitude [mV]	-15.82 ± 3.202	13	-21.1 ± 1.537	14	0.141

Supplementary Table 3: Electrophysiological properties of cortical pyramidal cells recorded in organotypic slice cultures from wildtype and heterozygous floxed *Scn1a*$^{+/A1783V}$ mice transduced with AAV8-Syn-Cre-GFP.

	**Wildtype**	*n*	**A1783V**	*n*	*p*
Resting membrane potential [mV]	-73.65 ± 1.398	15	-75.25 ± 1.28	25	0.424
Input resistance [MΩ]	263.6 ± 18.3	15	220.2 ± 11.32	25	0.056
Rheobase [pA]	46.67 ± 6.843	15	50 ± 6.61	25	0.742
Maximum firing frequency [Hz]	37.25 ± 3.172	15	31.7 ± 2.53	25	0.183
AP threshold [mV]	-44.18 ± 0.907	15	-43.58 ± 1.40	25	0.788
AP amplitude [mV]	69.01 ± 3.326	15	69.03 ± 3.82	25	0.997
AP rise time [ms]	1.279 ± 0.055	15	1.277 ± 0.06	25	0.987
AP half width [ms]	1.973 ± 0.147	15	1.972 ± 0.13	25	0.654
AP afterhyperpolarization amplitude [mV]	-8.953 ± 1.044	15	-8.895 ± 0.558	25	0.585

References

Alekov, A. K., Masmudur Rahman, M. D., Mitrovic, N., Lehmann-Horn, F., and Lerche, H. (2001). Enhanced inactivation and acceleration of activation of the sodium channel associated with epilepsy in man. *Eur. J. Neurosci.* 13, 2171–2176. doi:10.1046/j.0953-816X.2001.01590.x.
